# Substance P and Acute Pain in Patients Undergoing Orthopedic Surgery

**DOI:** 10.1371/journal.pone.0146400

**Published:** 2016-01-05

**Authors:** Barbara Lisowska, Katarzyna Siewruk, Aleksander Lisowski

**Affiliations:** 1 Department of Anaesthesiology, Medical Centre for Postgraduate Education Clinical Hospital Adam Gruca, CMKP Otwock, Poland; 2 Faculty of Veterinary Medicine, Warsaw University of Life Sciences, Warsaw, Poland; 3 Faculty of Production Engineering, Warsaw University of Life Sciences, Warsaw, Poland; Queen Mary University of London, UNITED KINGDOM

## Abstract

**Objective:**

There is a limited information about the role of Substance P (SP) in acute pain nociception following surgical stimulation in patients with a chronic inflammatory state not to mention the link between this neuropeptide level changes and intensity of pain. The goal of the research was to find the correlation between SP level changes and acute pain intensity in patients with rheumatoid arthritis undergoing elective orthopedic surgery.

**Material and Methods:**

Patients with rheumatoid arthritis (RA) were enrolled in the study. The correlation between acute pain intensity and concentration of SP in serum as well as in drainage fluid from postoperative wound was assessed in patients with RA who underwent Total Knee Replacement (TKA) under spinal anesthesia.

**Results:**

In patients with RA a correlation between intensity of acute pain and serum SP was found postoperatively, whereas there was no correlation between intensity of acute pain and concentration of SP in drainage fluid.

**Conclusions:**

1. The correlation between acute pain intensity and SP serum concentration was found postoperatively in patients with RA. 2. The correlation between acute pain intensity and SP concentration in drainage fluid was not found postoperatively in patients with RA.

## Introduction

A great deal of recent evidence suggests that SP and its receptors are involved in joint inflammation and associated with some aspects of the pathophysiology of RA [1], [2].

Total Knee Arthroplasty (TKA) is a useful surgical procedure for pain relief, which much improves the patient’s life comfort.

The acute postoperative pain is the direct consequence of the knee surgery following nociceptors excitation inside injured tissue.

There is a limited information describing the role of SP during acute nociception following surgical stimulation in patients with a chronic inflammatory state. Therefore, the correlation between the intensity of pain and systemic or local SP concentrations was studied in patients with RA who experienced an acute postoperative pain following orthopedic surgery.

The main objective of the research was to evaluate the link between intensity of acute pain in patients with RA undergoing elective orthopedic surgery and serum concentration of SP.

In addition, SP concentration in drainage fluid collected from postoperative wounds was measured.

## Materials and Methods

Patients with RA were randomly enrolled in the study.

All patients were diagnosed according to the criteria of the American Rheumatism Association [3].

Exclusion criteria included:

- Contraindications to spinal anesthesia (coagulation problems, infection at puncture site, neurological deficits, no patient’s consent for spinal)
- ASA physical status > III
- Severe liver or kidney diseases
- History of opioids dependence
- The presence of other inflammatory diseases

The intensity of acute pain in correlation with the concentration of SP in serum and drainage fluid from postoperative wound was examined postoperatively.

All patients underwent Total Knee Arthroplasty under spinal anesthesia. The classic cardiopulmonary parameters including ECG, BP (noninvasive blood arterial pressure), SpO_2_ (arterial blood oxygenation) were continuously monitored throughout surgery in all patients. None of the patients received opioids during surgery. For postoperative pain relief, the patients were given opioids and paracetamol. The venous blood samples were collected just before surgery (0 h) as well as 6 (6 h), 24 (24 h), 36 (36 h) hours postoperatively.

The disease activity was assessed with subjective and objective methods in all patients with RA. DAS 28 (Disease Activity Score) according to four variables with DAS 28 formula by DAS 28 Calculator v1.1-beta (Alfons & Michiel) was estimated.

The intensity of pain was evaluated using a numeric pain scale (NPS) from 0 to 5 where 0 corresponds with no pain, 1 –slight, 2 –moderate, 3 –intensive, 4 –severe and 5 –worst imaginable pain. The evaluation was done by patients.

The concentrations of SP in blood and drainage fluids samples were measured. The 4–5 ml venous blood samples were collected into the serum test- tubes, delivered to an analytical laboratory and centrifuged (2000 rpm/10 min). The sera were placed into 200 μl test- tubes and stored at -70°C. The 4–5 ml drainage fluid samples were collected postoperatively with a sterile syringe directly from postoperative wound drainage and the supernatants were aliquoted and stored the same as sera. The concentration of SP was measured with Substance P Immunoassay Test (R&D System) according to manual. The minimum detectable level for this assay was 8,0 pg/ml.

### Statistics

STATISTICA v.10 was used to carry out statistical analysis. For the tests performed at the time the repeated measures analysis was used. The sphericity assumption was estimated by Mauchly’s test. If the analysed variables did not correspond with the sphericity assumption then, for the result of the variance analysis, the more conservative Greenhouse-Geisser correction was used to adjust the degree of freedom associated with the F-value for repeated measure data the univariate and multivariate ANOVA tests were used. Correlation analyses were performed with the Spearman rank correlation test. Factor analysis was performed to find patterns among the measured variables. Factor loadings > 0.70 were considered. The factor analyses were performed with Varimax, with Kaiser normalisation as a rotation method. In order to identify interdependencies (their power was checked by the Snedecor F test) further tests were made to find a regression function, *P* values <0.05 were considered significant.

The protocol was approved by the medical Ethics Committee of Institute of Rheumatology in Warsaw. The experiments were conducted according to the principles expressed in the Declaration of Helsinki.

A written, informed consent was obtained from all patients.

## Results

The correlation between acute postoperative pain and SP serum was examined in 23 RA patients who underwent elective orthopedic surgeries. More women than men were enrolled in the study (22/1) at average age 59±10 years. The average RA disease duration was 21±8.7 years. The average DAS was 4.9±0.9, which indicates high disease activity. The SP drainage concentration (SP dr) and serum SP concentration were examined in 14 patients at the same time.

### Repeated measures analysis

Mauchly’s test indicated (Table 1) that the sphericity assumption for SP dr has been met χ^2^(5) = 8.63, *P* = 0.125, but for SP and NPS have been violated; χ^2^(9) = 51.03, *P* < 0.001 and χ^2^(5) = 13.33, *P* = 0.020 respectively. Because the sphericity assumption for SP dr has been fulfilled, then we can apply the one-dimensional approach. The analysis of variance results for repeated measures effects allow the conclusion that changes in SP dr subsequent measurements are statistically non significant (F = 0.64, *P* = 0.593). Again as the epsilon values are less than 0.80 (Table 1) thus more conservative Greenhouse-Geisser correction will be used for referring significance of the F ratio for interpreting results of SP and NPS (Table 2). There are statistically significant differences in SP and NPS at time (*P*-values less than 0.05).

**Table 1:** The results of the Mauchly’s test of sphericity for SP, SP dr and NPS.

Parameter	Effect	Mauchly’s W	Approx. χ^2^	df	*P*-value	Epsilon		
						Greenhouse-Geisser	Huynh-Feldt	Lower-bound
SP	time	0.08	51.03	9	<0.001^a^	0.459	0.499	0.250
SP dr	time	0.66	8.63	5	0.125			
NPS	time	0.53	13.33	5	0.020^a^	0.698	0.773	0.333

^a^ significant at 0.05 level.

**Table 2:** The results of the repeated measures univariate ANOVA test for SP and NPS taking into account the Greenhouse-Geisser correction adjustment.

Parameter	Correction	df	F	*P*-value
SP	Sphericity assumed	4	9.54^a^	<0.001
SP	Greenhouse-Geisser	1.837	9.54^a^	0.001
NPS	Sphericity assumed	3	10.63^a^	<0.001
NPS	Greenhouse-Geisser	2.093	10.63^a^	<0.001
Error (SP)	Sphericity assumed	88		
Error (SP)	Greenhouse-Geisser	40.414		
Error (NPS)	Sphericity assumed	66		
Error (NPS)	Greenhouse-Geisser	46.043		

^a^ significant at 0.05 level.

**Fig 1:**
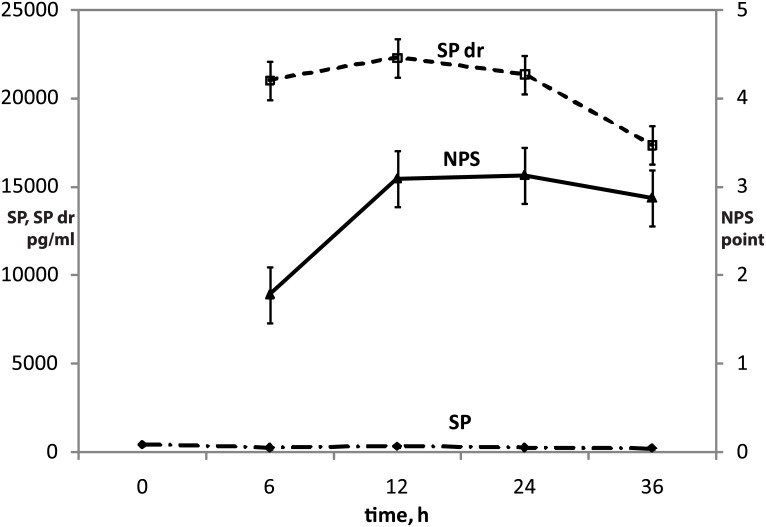
Changes of the serum concentration of substance P (SP) and SP drainage (SP dr) and the acute pain intensity NPS in time.

The unadjusted univariate method testing for a within-subject effect gives a little smaller *P*-values than the adjusted one if the sphericity condition is not satisfied. Instead of using the univariate method, we can confidently use the multivariate test for repeated measures data as it does not require a specific covariance structure. Multivariate tests—Pillai’s Trace, Hotelling’s Trace, Wilk’s Lambda and Roy’s Largest Root confirmed the previous results in terms of statistically significant differences (*P* << 0.05) of repeated measures of SP and NPS in time (Table 3). Changes of the serum concentration of substance P (SP) and SP drainage (SP dr) and the acute pain intensity NPS in time were presented graphically on Fig 1.

**Table 3:** The results of the multivariate ANOVA tests for SP and NPS.

Parameter	Effect	Test	Value	F	Hypothesis df	Error df	*P*-value
SP	time	Wilks' Lambda	0.473	5.30	4	19	0.005
SP	time	Pillai's Trace	0.527	5.30	4	19	0.005
SP	time	Hotellng's Trace	1.115	5.30	4	19	0.005
SP	time	Roy's Largest Root	1.115	5.30	4	19	0.005
NPS	time	Wilks' Lambda	0.437	8.60	3	20	0.001
NPS	time	Pillai's Trace	0.563	8.60	3	20	0.001
NPS	time	Hotellng's Trace	1.291	8.60	3	20	0.001
NPS	time	Roy's Largest Root	1.291	8.60	3	20	0.001

In order to find out if the values measured at the time change with respect to the input values, an analysis of contrasts was done (Table 4).

**Table 4:** The results of the contrasts analysis for SP and SP dr and NPS.

Variable	SS	df	MS	F	*P*-value
SP	354133.2	1	354133.2	10.28^a^	0.004
Error (SP)	758189.3	22	34463.2		
SP dr	7.9E+06	1	7862907	0.06	0.806
Error (SP dr)	2.8E+09	22	127833891		
NPS	12.6	1	12.55652	7.84^a^	0.010
Error (NPS)	35.2	22	1.60198		

^a^ significant at 0.05 level.

The difference between SP value measured before the operation and SP values measured in the next hours after the surgery was important (*P* = 0.004). In postoperative NPS values we observed a difference between the first measurement and the following ones (P = 0.010). For SP dr however this difference was not statistically significant (P = 0.806).

### Correlation between NPS, SP, SP dr and postoperative time

Spearman rank correlations between the NPS, SP, SP dr and postoperative time were calculated (Table 5). A weak correlation (0.219) between NPS and time, and a weak negative correlation (-0.224) between SP and time, and a good correlation (0.504) between NPS and SP were found.

**Table 5:** The results of analysis of correlation between postoperative time, NPS, serum SP (SP) and SP drainage (SP dr).

Parameter	Time	NPS	SP	SP dr
Time	1.000			
NPS	0.219^a^	1.000		
SP	-0.224^a^	0.504^a^	1.000	
SP dr	-0.056	0.085	0.021	1.000

^a^ correlation with *P* < 0.05.

Factor analysis performed on the RA group yielded two factors with eigenvalues > 1, explaining 68% of total variation between the measured variables (Table 6). NPS, and SP were grouped together and time and SP dr (but with factor loading >0.5) created another group.

**Table 6:** Factor analysis on postoperative time, NPS, serum SP (SP) and SP drainage (SP dr).

Variable	Factor	
	1	2
Time		-0.854
NPS	0.840	
SP	0.873	
SP dr		0.538

Great differences were found between serum SP and SP drainage values. The SP drainage concentration was hundred times higher than SP serum concentration. Nevertheless, the mean pain severity value was 2.6.

Having found correlation between the variables an attempt to extract a functional dependence was conducted and a regression analysis for this purpose was performed (Table 7). Based on the results of critical level of significance, a statistical significance is confirmed in relation to the values of pain the concentration of logarithmic SP—ln(SP) (*P* < 0.001) at a given time (*P* = 0.008).

**Table 7:** The results of regression analysis for acute pain intensity according to NPS depends on the postoperative time and the logarithmic concentration of substance P—ln(SP) and the concentration of SP in drainage fluid.

Parameter	*β*	Standard error of *β*	*b*	Standard error of *b*	t(48)	*P*-value
Intercept			-1.770	0.992	-1.784	0.087
Time	0.334	0.120	0.038	0.014	2.779	0.008
Ln(SP)	0.482	0.120	0.712	0.177	4.025	<0.001
SP dr	0.014	0.120	0.000	0.000	0.117	0.907

Regression summary for dependent variable NPS: correlation coefficient R = 0.5619, determination coefficient R^2^ = 0.3158, adjusted correlation coefficient R^2^ = 0.2730, values of the Fisher-Snedecor test F(3, 48) = 7.38, critical level *P*-value *P* < 0.001, standard error of estimate: 1.11.

The concentration of substance P in the drainage fluid did not have a statistically significant effect on the intensity of the pain after the operation, because the values of the critical level of significance and intercept are respectively *P* = 0.907 and *P* = 0.087. Therefore, using the module forward stepwise regression, the latter two quantities have been removed, and the results of the regression analysis are shown in Table 8. In this case, both values are statistically significant and the regression equation overall rating is very high, as evidenced by an adjusted coefficient of determination R^2^ = 0.8499. However, it cannot be directly compared with the previous value, because in this case has been removed from the equation, moving the graph up the value. Based on the test regression analysis (Tables 7 and 8) it can be concluded that the value of NPS is connected with both the time and SP and the following relationship can be drawn.

$$NPS_{RA}=0.03t+0.41\text{ln}(SP).$$

**Fig 2:**
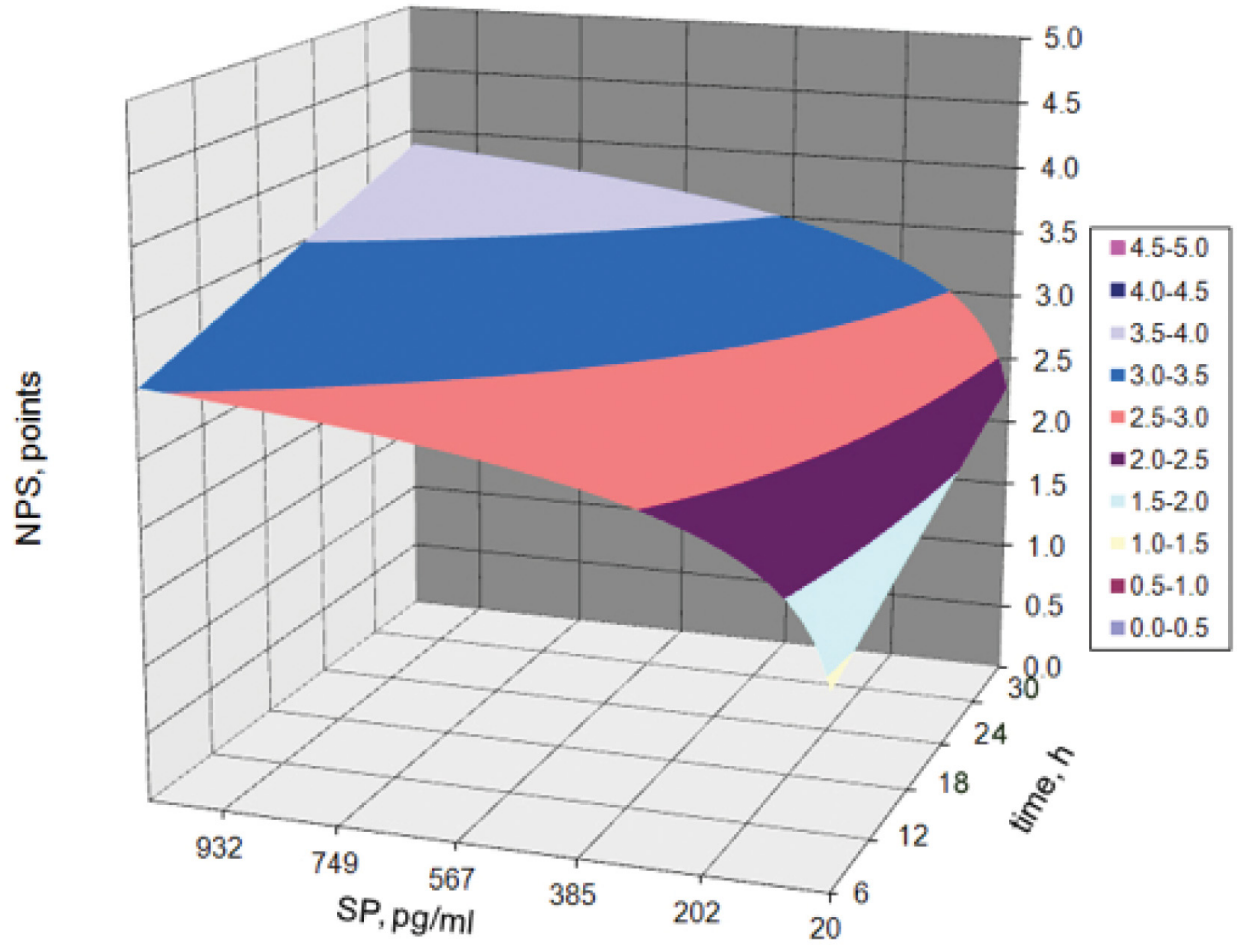
The interaction between the serum concentration of substance P (SP) and the acute pain intensity according to NPS and time.

Graphical interpretation of the formula at Fig. Fig 2. The interaction between the serum concentration of substance P (SP) and the acute pain intensity according to NPS and time was presented.

**Table 8:** The results of regression analysis for acute pain intensity according to NPS depends on the postoperative time and the logarithmic concentration of substance P—ln(SP) after removal of the concentration of SP in fluid drainage.

Parameter	*β*	Standard error of *β*	*b*	Standard error of *b*	t(87)	*P*-value
Time	0.223	0.098	0.029	0.013	2.273	0.027
Ln(SP)	0.730	0.098	0.409	0.055	7.450	<0.001

Regression summary for dependent variable NPS: correlation coefficient R = 0.9249, determination coefficient R^2^ = 0.8554, adjusted correlation coefficient R^2^ = 0.8496, values of the Fisher-Snedecor test F (2, 50) = 147.86, critical level *P*-value *P* < 0.001, standard error of estimate: 1.12.

Based on the graph of the surface (Fig 2) it can be seen that immediately after the operation the patients with small values of intensity of substance P (SP) experienced low intensity pain, but those with maximum concentration of substance P (1200 pg/ml) complained of severe pain at the level of 4 points. The dynamics of increase in pain perceived by the patients was higher with the increase of the intensity of substance P than with the passage of time after the operation, especially for small values of the maximum intensity of substance P. Patients experienced maximum pain in the last hour of the monitored operation (36 h), which was 2.0–2.5 points with patients with the lowest intensity values of substance P to 4 points for patients with the highest intensity values of the substance P (about 1200 pg/ml).

As before, for chronic pain in RA patients with acute pain, there is a clear change in the dynamics of pain perceived by patients who had substance P concentrations above 200 pg/ml. Below this value, the concentration of substance P (200 pg/ml) of the dynamics of change in pain intensity was high, and was 0.25 points out of 100 pg/ml, and after exceeding a value of 200 pg/ml the increase of pain was reduced almost twice in the value of 0125 points in 100 pg/ml.

At 36 h after surgery patients complained of more severe pain (2.5–3.0 points) even at low values of the concentration of substance P and were more sensitive to it. Along with increasing concentrations of substance P in a smaller range of values, for the value of 200 pg/ml the intensity of the pain reached the level of 3 points. For SP value about 380 pg/ml, the pain was already estimated at 3.5 point. These differences can be observed on the width of the contour lines in the Fig 2. The interaction between the serum concentration of substance P (SP) and the acute pain intensity according to NPS and time, which corresponds to a graduated scale of 0.5 points.

## Discussion

The high preoperative serum concentration of SP in all studied patients was found. However, it is directly related with chronic, advanced, ongoing, auto immunological disease.

The link between serum concentration of SP and the intensity of acute pain postoperatively was assessed. Therefore, significant correlation between the intensity of acute pain and serum SP level changes in RA patients in accordance with the results given by Michaels et al. was found [4] In contrast to our study, the results concerned children with sickle cell disease (SCD) who suffer from an acute vaso-occlusive pain. They showed the high level of SP in serum, and similarly to us, they confirmed the increase of SP concentration during critical pain. In conclusion, they also suggested a therapeutic potential of neurokinin receptor antagonists in the treatment of pain.

The correlation between the postoperative concentration of plasma SP and the labor pain among pregnant and non-pregnant healthy women enrolled in the study by Conversely, Dalby et al. [5] were not confirmed.

Additionally, different factors combined with nociceptive stimulation could have an influence on serum SP concentration. The type of anesthesia is one of them. The systemic SP concentration mainly depends on neuronal nociceptive transmission, which is blocked by spinal anesthesia. However, the SP concentration in drainage fluid depends on inflammatory mediators release triggered by mastocytes degranulation. As the immediate result of the sensory blockade, the SP concentration increase and respectively IL-10 decrease was found postoperatively in wound surrounding tissue as the example of imbalance among pro- and anti-inflammatory components [6].

The spinal anesthesia with bupivacaine does not seem to influence the concentration of SP in serum or wound fluid. Although the results reported by Carvalho et al. show that the local application of bupivacaine resulted in an increased concentration of SP wound exudates [7].

Knee joint replacement as a major orthopedic surgery causes significant postoperative pain that can be relieved by opioids administration.

The postoperative measurement of average consumption of opioids was done additionally. Nevertheless, the long-term morphine analgesia might influence SP concentration. The postoperative analgesia was provided with morphine at a dose 10 mg or pethidine at a dose 1 mg/kg administered subcutaneously every 4–6 hrs. Additionally, 1 g of paracetamol intravenously three times per day for all the patients was administrated. None of the patients received intrathecal opioids and tramadol IV/IM. The mean morphine consumption was about 70 mg MF and 350 mg pethidine during the 36—hour study period. This treatment is optimal for effective pain relief. It is known that opioids cause the presynaptic attenuation of neurotransmitter release from sensory neurons [8]. The subcutaneous acute morphine administration may also induce matrix metalloproteinases 9 (MMP 9) up regulation in dorsal root ganglia (DRG) neurons [9]. MMP 9 is an enzyme with the ability of degradation of tachykinin peptides such as SP, neurokinin A, neurokinin B [10]. Therefore, it seems that acute morphine treatment influences the decrease of the SP level via MMP 9 in dorsal root ganglia. No studies about the influence of acute morphine treatment on serum concentration of SP were found.

On the other hand, there was not any correlation between the SP concentration in drainage fluid and postoperative acute pain severity. The SP concentration in drainage fluid however was hundredfold higher than in the serum. Sahbaie et al. have reported that the high concentration of SP in drainage may contribute to the development of hyperalgia or allodynia in the skin surrounding the wound [11]. None of our patients however suffered from allodynia or hyperalgesia during hospitalization or 14 days follow up time. The extremely high level of SP in drainage fluid is an advantage of the local response over the systemic response what was confirmed by our previous study [12]. The SP released into wound tissue acts directly like a neurotransmitter and indirectly like a stimulator of pro inflammatory cytokines (IL-1, IL-6 and TNF) released from monocytes/macrophages. Therefore, the correlation between concentration of SP in drainage fluid and intensity of acute pain was assessed [13].

The next factor that could have influenced the level SP drainage was the use of a tourniquet to prevent bleeding during surgery. SP is known as one of many pro inflammatory components in reperfusion injury due to tourniquet ischemia [14]. The time of using tourniquet was less that 2 h (mean 93 min) so that the histological changes in tissues were not expected nonetheless the functional changes connected with releasing many mediators. Moreover, in our study, the presence of prostheses could also have influenced the results obtained on SP drainage. There were no publications presenting the link between prosthesis and SP serum concentration. It seems significant that the observation time was too short to affect the presence of prostheses.

The administration of cytokine antagonist is beneficial in RA therapy. The multimodal action of NK-1 and NK-2 receptors allows to use their antagonists in the therapy of different disorders moreover the use of the SP antagonist might be beneficial in degenerative joint disease therapy. The anti nociceptive action of the substance P antagonists was confirmed to be beneficial in therapy of acute pain because it inhibits the development of opioid anti nociceptive tolerance [15], [16].

The new opioid peptide analogue, which acts as an agonist for opioid receptors as well as an antagonist for the NK1 receptor by Matalinska et al., was found. Additionally, its anti proliferative properties were confirmed which is advantageous in the cancer pain therapy [17].

The correlation between acute pain and SP serum concentration is a new approach for further research about the usefulness of NK-1 receptor antagonists or SP as a new kind of analgesics, which need to be proven.

## Conclusions

1. The correlation between acute pain intensity and SP serum concentration was found postoperatively in patients with RA.
2. The correlation between acute pain intensity and SP concentration in drainage fluid was not found postoperatively in patients with RA.
